# Polatuzumab Vedotin Induced CD20 Upregulation Contributes to the Efficacy of Mosunetuzumab in Combination With Polatuzumab Vedotin in Diffuse Large B‐Cell Lymphoma Preclinical Models

**DOI:** 10.1002/jha2.70169

**Published:** 2025-10-24

**Authors:** Natsumi Kawasaki, Sei Shu, Mayu Tomita, Xiaoxiao Liu, Shigeki Yoshiura, Yoriko Yamashita‐Kashima

**Affiliations:** ^1^ Product Research Department Chugai Pharmaceutical Co., Ltd Yokohama Kanagawa Japan

**Keywords:** CD20, DLBCL, mosunetuzumab, non‐Hodgkin lymphoma, polatuzumab vedotin

## Abstract

**Background:**

Aggressive non‐Hodgkin lymphoma (aNHL) often relapses after first‐line treatment. Clinical data supports the safety and efficacy of the combination of mosunetuzumab, a CD20×CD3 bispecific antibody, and polatuzumab vedotin, an anti‐CD79b antibody drug conjugate (Mosun‐Pola) in relapsed/refractory aNHL. This study investigated the molecular mechanism behind the combination effect of Mosun‐Pola in human diffuse large B‐cell lymphoma (DLBCL) cell lines.

**Methods:**

The in vitro Mosun‐Pola efficacy in DLBCL cells (SU‐DHL‐8 and HT) was evaluated by T cell‐dependent cellular cytotoxicity (TDCC) assay. CD20‐stable‐knockdown SU‐DHL‐8 cells were established using lentiviral short hairpin RNA. Surface and T‐cell activation marker proteins expression were determined by flow cytometry. Human T‐cell‐injected mice or humanized NOD/Shi‐scid, IL‐2Rγnull (huNOG) mice were used for an in vivo study.

**Results:**

An in vitro TDCC assay showed a synergistic effect in SU‐DHL‐8 and HT cells. Based on our experimental results of suppressing CD20 expression, it was suggested that this combination effect could be caused by an increase in CD20 expression by polatuzumab vedotin. In addition, examining the effects of CD20 upregulation in tumor cells on T‐cell activation demonstrated that the combination of Mosun‐Pola enhanced T‐cell activation markers in both CD4+ and CD8+ T cells during the TDCC reaction. In vivo studies, using human immune system‐reconstituted mouse models confirmed that polatuzumab vedotin enhanced CD20 expression in tumors, and the combination of Mosun‐Pola showed significantly improved anti‐tumor effects compared with single‐drug treatments.

**Conclusion:**

These findings suggest that polatuzumab vedotin‐induced CD20 upregulation provides a molecular rationale to explain the synergistic effect of this combination therapy.

**Trial Registration:**

The authors have confirmed clinical trial registration is not needed for this submission.

## 1 Introduction

Aggressive non‐Hodgkin lymphoma (aNHL) is a heterogeneous group of tumors, with diffuse large B‐cell lymphoma (DLBCL) being the most common type [1]. Whilst the majority of aNHL cases are curable, 20%–40% of patients experience relapse or become refractory to front‐line treatment [1, 2]. Years of research have led to an evolution in treatment options for patients with relapsed/refractory (R/R) aNHL. These advancements include the development of targeted therapies such as CD19‐directed chimeric antigen receptor T‐cell therapy [3, 4, 5], the anti‐CD19 monoclonal antibody tafasitamab [6] and the anti‐CD79b antibody drug conjugate (ADC) polatuzumab vedotin [7, 8]. In addition, regimens including CD20×CD3 T‐cell‐engaging bispecific antibodies have been developed or approved in recent years and have demonstrated promising efficacy [9, 10, 11].

Mosunetuzumab is a full‐length, humanized immunoglobulin G1–based CD20×CD3 bispecific antibody that engages and redirects T cells to eliminate malignant B cells [10]. The efficacy and safety of mosunetuzumab in NHL has been studied in a single‐arm, phase 2 study (NCT02500407) [12, 13]. Based on the results of this study, mosunetuzumab was recently approved in several countries for R/R follicular lymphoma in patients previously treated with two or more different systemic therapies. Polatuzumab vedotin is an ADC that targets CD79b, a transmembrane protein expressed on the surface of B cells [7, 8]. After binding to CD79b, polatuzumab vedotin is internalized and releases a potent microtubule inhibitor, monomethyl auristatin E. Polatuzumab vedotin has been approved for the treatment of previously untreated or R/R DLBCL based on the results of the phase 3 POLARIX study (NCT03274492) [14] and a phase 1b/2 study (NCT02257567), respectively [15].

In recent years, the development of combination therapies involving CD20×CD3 bispecific antibodies for the treatment of lymphoma has advanced, and the combination of mosunetuzumab plus polatuzumab vedotin (Mosun‐Pola) is one of the promising regimens. The results of a phase 1b/2 study (NCT03671018) demonstrated that Mosun‐Pola is an effective therapeutic approach to achieve durable responses with a manageable safety profile in patients with R/R aggressive large B cell lymphoma [16]. Based on these results, a global, randomized, open‐label, multicentre, Phase 3 study (SUNMO: NCT05171647) evaluating Mosun‐Pola in R/R NHL is ongoing [17].

In our previous research, we demonstrated that polatuzumab vedotin enhances the surface expression of CD20 protein in some polatuzumab vedotin‐refractory cell lines through activation of ERK and AKT signaling pathways [18]. In that same study, we reported that polatuzumab vedotin treatment upregulates sensitivity to rituximab‐induced complement‐dependent cytotoxicity (CDC) and antibody‐dependent cellular cytotoxicity (ADCC) sensitivity, exhibiting significant synergistic anti‐tumor effects both in vitro and in vivo. Therefore, we hypothesized that the upregulation of CD20 expression induced by polatuzumab vedotin might also enhance the efficacy of mosunetuzumab to redirect T cells. In this study, we investigated whether the upregulation of CD20 expression by polatuzumab vedotin contributes to the combination effect with mosunetuzumab and explored the rationale and mechanisms behind the combination of these two drugs.

## 2 Materials and Methods

### 2.1 Reagents

Mosunetuzumab was obtained from F. Hoffmann‐La Roche and polatuzumab vedotin was obtained from Chugai Pharmaceutical. Human immunoglobulin G (HuIgG) was purchased from MP Biomedicals, MK‐2206 2HCl was purchased from Selleck Chemicals and U0126 was purchased from Promega.

### 2.2 Cell Cultures

SU‐DHL‐8 and HT cells were obtained from the American Type Culture Collection (ATCC). These cells were maintained in RPMI‐1640 ATCC modification (Thermo Fisher Scientific) with 10% fetal bovine serum (Corning). All cells were cultured in a humidified atmosphere of 5% CO_2_ at 37°C.

### 2.3 T‐Cell‐dependent Cellular Cytotoxicity (TDCC) Assay

Human peripheral blood mononuclear cells (PBMCs) were purchased from STEMCELL Technologies. Human T cells from PBMCs were purified using an EasySep Human T Cell Isolation Kit (STEMCELL Technologies). Target cells were labeled using CellTrace Violet Cell Proliferation Kit (Thermo Fisher Scientific) and seeded, then mosunetuzumab, polatuzumab vedotin, and effector T cells were added to a target ratio of 5:1. After incubation, dead target cells were stained using Fixable Viability Dye (FVD) (Thermo Fisher Scientific). Target‐specific cell killing was detected by FVD staining in target cells by flow cytometry. The evaluation of the synergistic combination effect was conducted using the Bliss independence model [19, 20].

### 2.4 Lentivirus Infection

Control or human CD20 small hairpin (sh)RNA lentivirus was purchased from VectorBuilder. The shRNA target sequence was scramble controlled: CCTAAGGTTAAGTCGCCCTCG; MS4A1 (CD20): GAGACATGCTGACTTTCATTT. SU‐DHL‐8 cells were infected with lentivirus for one day (multiplicity of infection >10). Cells were cultured in medium containing 500 µg/mL Geneticin Selective Antibiotic (G418 Sulfate) (Thermo Fisher Scientific) for the selection of stable clones.

### 2.5 T‐Cell‐Injected Mouse Model

In the T‐cell‐injected model, female NOD/ShiJic‐scidJcl (NOD/SCID) mice aged 5 weeks were purchased from CLEA Japan. Each mouse was inoculated subcutaneously (SC) with 5 × 10^6^ cells of each cell line. When the tumor was established, mice were randomized by tumor volume and assigned to indicated study groups. To ensure the T cells engraft in mice, anti‐asialo GM1 (FUJIFILM Wako Pure Chemical Corporation) was administered intraperitoneally (IP) on Day 0. Human T cells were purified using an EasySep Human T Cell Isolation Kit from PBMCs and expanded by culturing using Dynabeads Human T‐Activator CD3/CD28 for T Cell Expansion and Activation (Thermo Fisher Scientific). Expanded human T cells (3 × 10^7^ cells/mouse) were injected IP on Day 1. Control HuIgG or polatuzumab vedotin were administered intravenously (IV) on Day 1. Control HuIgG or mosunetuzumab were administered IV on Days 1, 8, and 15. To evaluate the anti‐tumor activity of the test agents, tumor volume was evaluated as described previously [21].

### 2.6 Humanized NOD/Shi‐Scid, IL‐2Rγnull (huNOG) Mouse Model

To establish the huNOG model, female NOG mice aged 6 weeks were purchased from CLEA Japan. Mice were irradiated (2.5 Gy; MBR‐1520R‐3; Hitachi Power Solutions) one day before transplantation of human CD34^+^ cells (Lonza) via IV injection (0.9 × 10^5^ cells/mouse). After chimerism analysis, HT cells (5 × 10^6^ cells per mouse) were then inoculated SC into mice. After inoculation, mice were randomized by tumor volume and assigned to indicate study groups. Control HuIgG or polatuzumab vedotin were administered IV on Day 1. Control HuIgG or mosunetuzumab were administered IV on Days 1, 8, and 15. To evaluate the anti‐tumor activity of the test agents, tumor volume was evaluated as described previously [21].

### 2.7 Flow Cytometry

For in vivo studies, tumor samples were digested using Tumor Dissociation Kit human (Miltenyi Biotec). Cells prepared either in vitro or in vivo were stained with indicated antibodies. Anti‐CD20 (Clone: 2H7), anti‐CD69 (Clone: FN50), anti‐CD19 (Clone: HIB19), anti‐CD4 (Clone: RPA‐T4) and anti‐CD8 (Clone: HIT8α) antibodies, and IgG1κ (Clone: MOPC‐21) and IgG2bκ (Clone: 27‐35) isotype controls were obtained from BD Biosciences. Stained cells were analyzed by BD LSRFortessa X‐20 (BD Biosciences) and FlowJo v10.8.1 software (BD Biosciences). Geometric mean fluorescence intensity (gMFI) was normalized by subtracting the gMFI value of the respective isotype control‐stained cells from the observed gMFI of labeled cells.

### 2.8 Ethical Statement

All animal experiments were reviewed and approved by the Institutional Animal Care and Use Committee at Chugai Pharmaceutical Co., Ltd., which is an institute accredited by AAALAC international, and conformed to the Guide for the Care and Use of Laboratory Animals published by the Institute for Laboratory Animal Research.

### 2.9 Statistical Analysis

Student's *t*‐test was used for comparisons between two groups. One‐tailed, one‐sample *t*‐tests were used to determine whether Delta Bliss scores were significantly greater than zero. Dunnett's test was used for multiple comparisons with the control group and Tukey's honest significant difference test was used for multiple comparisons within groups. For tumor growth experiments, *p*‐values for the Wilcoxon rank sum test were adjusted by the Holm–Bonferroni method. All statistical analyses were performed using JMP 17.2.0 software (JMP Statistical Discovery).

## 3 Results

### 3.1 Synergistic Effects of Mosun‐Pola in Human DLBCL Cell Lines

**FIGURE 1:**
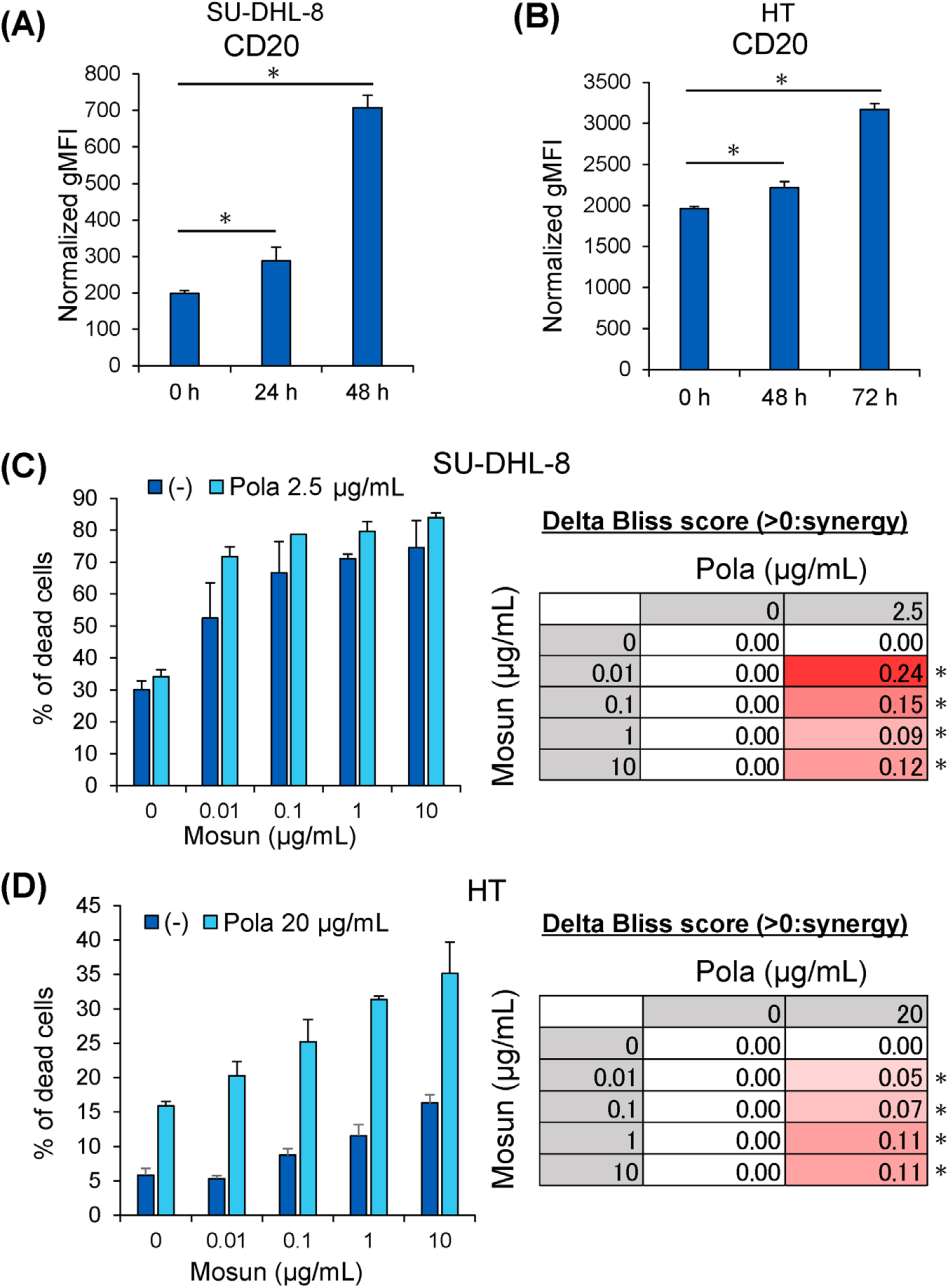
Investigating the combination effects of Mosun‐Pola by in vitro TDCC assay. (A) SU‐DHL‐8 cells were treated With polatuzumab vedotin (2.5 µg/mL) for indicated hours. Cell surface expression of CD20 was measured by flow cytometry. Normalized gMFI was calculated by subtracting the fluorescence of the isotype. Mean values + SD, *n *= 3. *p < 0.05 by Dunnett's test compared to 0 h. (B) HT cells were treated With polatuzumab vedotin (20 µg/mL) for indicated hours. Cell surface expression of CD20 was measured by flow cytometry. Normalised gMFI was calculated by subtracting the fluorescence of the isotype. Mean values + SD, *n *= 3. *p < 0.05 by Dunnett's test compared to 0 h. (C) SU‐DHL‐8 cells were pretreated With polatuzumab vedotin (2.5 µg/mL) for 24 h. Efficacy of Mosun‐Pola was evaluated by TDCC assay. Cytotoxicity was assessed 24 h after the initiation of TDCC. Mean values + SD, *n *= 3. The Delta Bliss score is shown using the mean value at each concentration. Statistical significance was assessed using one‐tailed, one‐sample *t‐*tests (upper‐tail) against the null hypothesis that Delta Bliss score equals zero. *p < 0.05. (D) HT cells were pretreated with polatuzumab vedotin (20 µg/mL) for 48 h. Efficacy of Mosun‐Pola was evaluated by TDCC assay. Cytotoxicity was assessed 24 h after the initiation of TDCC. Mean values + SD, *n *= 3. The Delta Bliss score is shown using the mean value at each concentration. Statistical significance was assessed using one‐tailed, one‐sample *t*‐tests (upper‐tail) against the null hypothesis that Delta Bliss score equals zero. *p < 0.05 gMFI, geometric mean fluorescence intensity; Mosun, mosunetuzumab; Pola, polatuzumab vedotin; SD, standard deviation; TDCC, Tcell‐dependent cellular cytotoxicity.

To investigate the combination effects of Mosun‐Pola, we used two germinal center B‐cell‐like (GCB) type human DLBCL cell lines, SU‐DHL‐8 and HT cells, which were refractory to polatuzumab vedotin. These cell lines were selected because we have previously shown that polatuzumab vedotin increases CD20 expression within them [18]. First, we treated cells with polatuzumab vedotin under concentrations consistent with previous reports and evaluated its effect on CD20 expression by flow cytometry. As a result, it was confirmed that polatuzumab vedotin significantly enhances the expression of CD20 on the cell membrane surface in both SU‐DHL‐8 and HT cells as previously described (Figure 1A,B). Subsequently, under conditions where the upregulation of CD20 expression by polatuzumab vedotin was observed, the effect of combining mosunetuzumab with polatuzumab vedotin was evaluated using an in vitro TDCC assay. The combination effect was assessed using the Delta Bliss score with a positive score indicating a synergistic effect. Mosun‐Pola showed a synergistic effect under several concentrations of mosunetuzumab in SU‐DHL‐8 cells (Figure 1C). Similarly, a synergistic effect was also demonstrated in the HT DLBCL cell line (Figure 1D).

### 3.2 Influence of Polatuzumab Vedotin‐Induced CD20 Upregulation on Mosunetuzumab‐Mediated Cytotoxicity

**FIGURE 2:**
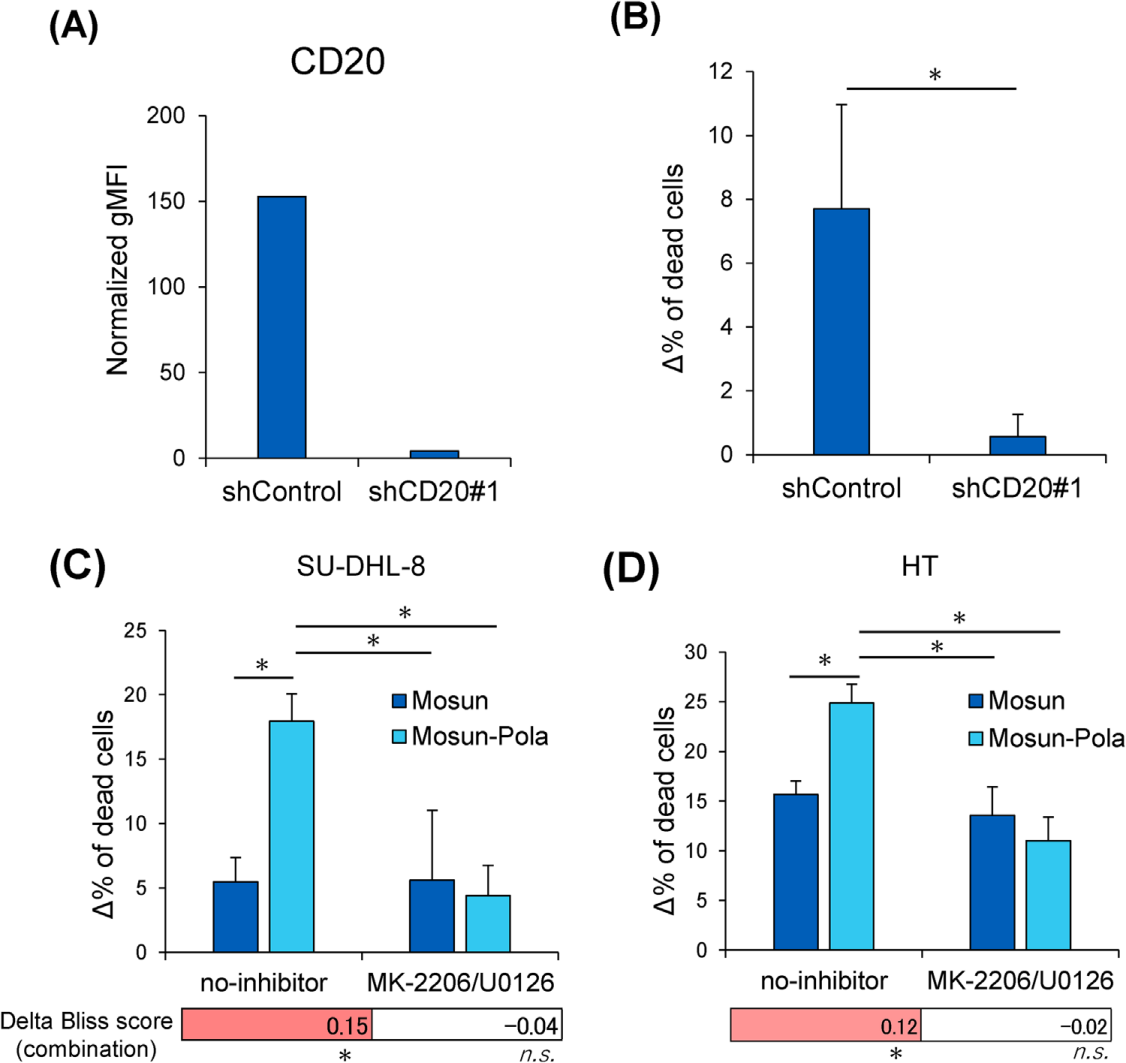
Investigation of the effects of CD20 upregulation by polatuzumab vedotin on sensitivity to mosunetuzumab. (A) Surface expression of CD20 on SU‐DHL‐8 cells transduced with either CD20‐targeting shRNA (shCD20#1) or non‐targeting shRNA (shControl) was measured by flow cytometry. Normalized gMFI was calculated by subtracting the fluorescence of the isotype. (B) Mosunetuzumab sensitivity was evaluated by TDCC assay (mosunetuzumab: 0.01 µg/mL) for 24 h. The graph shows the values obtained by subtracting the Mosun 0 µg/mL value under each condition. Mean values + SD, *n *= 3. *p < 0.05 by Student's *t‐*test. (C) SU‐DHL‐8 cells were pretreated With MK‐2206 (0.5 µM) and U0126 (10 µM) for 24 h, followed by addition of polatuzumab vedotin (2.5 µg/mL) for another 24 h. The combination effect of Mosun‐Pola was then evaluated by TDCC assay for 24 h (mosunetuzumab: 0.01 µg/mL). The graph shows the values obtained by subtracting the mosunetuzumab 0 µg/mL value under each condition. Mean values + SD, *n *= 3. *p < 0.05 by Tukey's HSD test. The Delta Bliss score is shown using the mean value at each concentration. Statistical significance of the Delta Bliss scores was assessed using one‐tailed, one‐sample *t*‐tests (upper‐tail) against the null hypothesis that the mean Delta Bliss score equals zero. *p < 0.05. (D) HT cells were pretreated with MK‐2206 (0.5 µM) and U0126 (10 µM) with or without polatuzumab vedotin (20 µg/mL) for 24 h. Cells were then washed and treated with polatuzumab vedotin (20 µg/mL) for another 24 h. The effect of Mosun‐Pola was then evaluated by TDCC assay for 24 h (mosunetuzumab: 0.1 µg/mL). The graph shows the values obtained by subtracting the mosunetuzumab 0 µg/mL value under each condition. Mean values + SD, *n *= 3. *p < 0.05 by Tukey's HSD test. The Delta Bliss score is shown using the mean value at each concentration. Statistical significance of the Delta Bliss scores was assessed using one‐tailed, one‐sample *t*‐tests (upper‐tail) against the null hypothesis that the mean Delta Bliss score equals zero. *p < 0.05. gMFI, geometric mean fluorescence intensity; HSD, honest significant difference; Mosun, mosunetuzumab; n.s, not significant; Pola, polatuzumab vedotin; SD, standard deviation; TDCC, T‐cell–dependent cellular cytotoxicity.

To determine if CD20 expression levels influence sensitivity to mosunetuzumab, we established SU‐DHL‐8 cells with stable CD20 knockdown by introducing shRNA targeting CD20 (shCD20#1). We confirmed the knockdown of CD20 expression in these cells by flow cytometry (Figure 2A and Figure S1). We then examined the sensitivity to mosunetuzumab by in vitro TDCC assay and found that shCD20#1 cells showed reduced sensitivity to mosunetuzumab monotherapy compared with control cells (Figure 2B), suggesting that adequate CD20 expression is essential for mosunetuzumab efficacy in these cells. We previously reported that polatuzumab vedotin induces CD20 upregulation through AKT and ERK signaling [18]. Therefore, we conducted analyses using an AKT inhibitor (MK‐2206) and a MEK inhibitor (U0126) to suppress these signaling pathways. We confirmed that polatuzumab vedotin‐induced upregulation of CD20 expression was attenuated by AKT and ERK inhibitors (Figure S2A,B). Under these conditions, the synergistic effect of Mosun‐Pola in vitro was shown to be attenuated, as demonstrated by in vitro TDCC assay in both SU‐DHL‐8 and HT cells (Figure 2C,D). These findings suggest that polatuzumab vedotin‐induced CD20 upregulation through AKT and ERK signaling may contribute to the synergistic effects of Mosun‐Pola.

**FIGURE 3:**
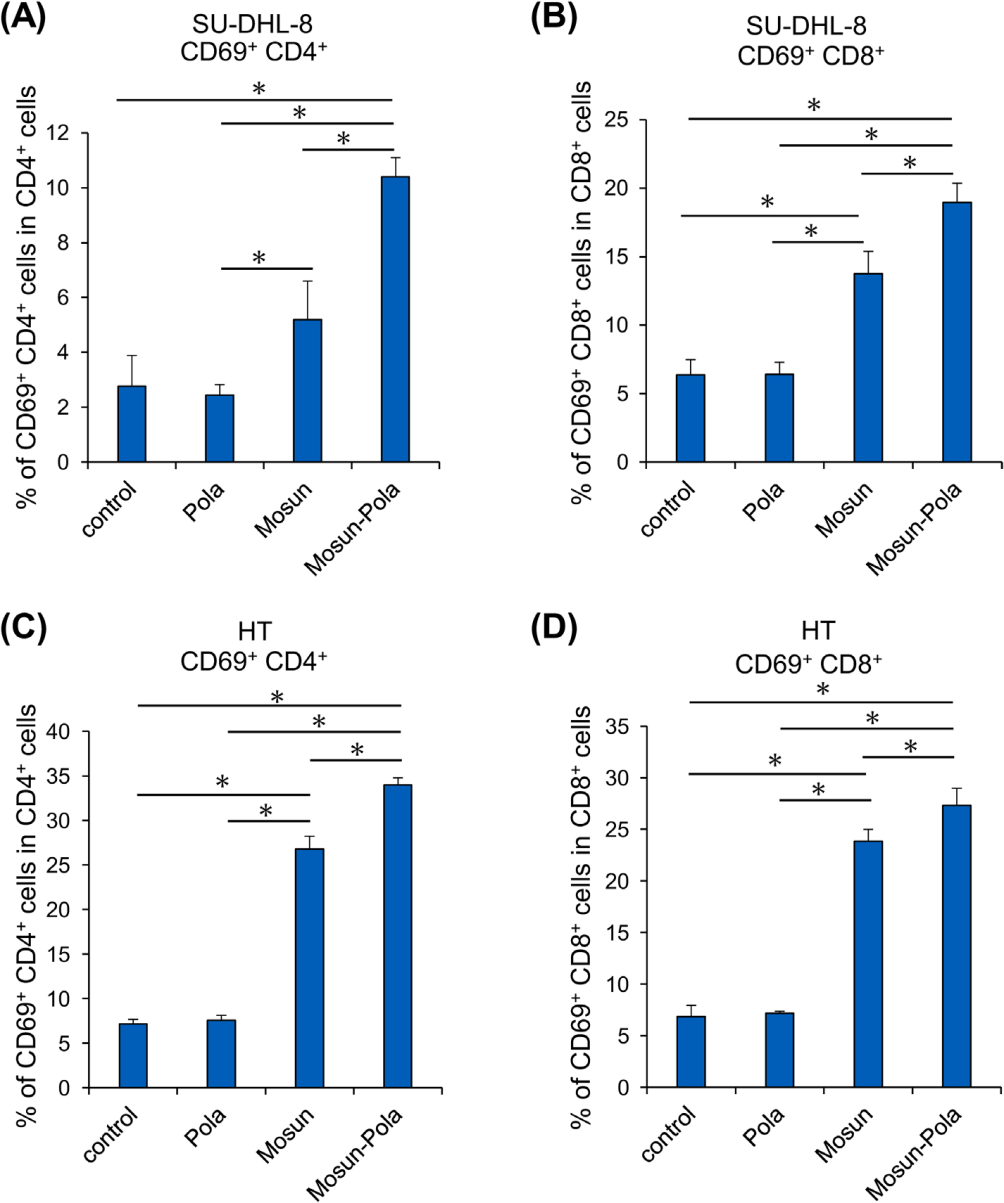
Effects of Mosun‐Pola combination on T‐cell activation. (A, B) SU‐DHL‐8 cells were pretreated with polatuzumab vedotin (2.5 µg/mL) for 48 h. The percentage of CD69^+^ CD4^+^ cells (A) and CD69^+^ CD8^+^ cells (B) was assessed 4 h after the initiation of TDCC (mosunetuzumab: 0.01 µg/mL). Mean values + SD, *n *= 3. *p < 0.05 by Tukey's HSD test. (C, D) HT cells were pretreated with polatuzumab vedotin (20 µg/mL) for 72 h. The proportion of CD69^+^ CD4^+^ cells (C) and CD69^+^ CD8^+^ cells (D) was assessed 4 h after the initiation of TDCC (mosunetuzumab: 0.1 µg/mL). Mean values + SD, *n *= 3. *p < 0.05 by Tukey's HSD test. HSD, honest significance test; Mosun, mosunetuzumab; Pola, polatuzumab vedotin; SD, standard deviation; TDCC, T‐cell–dependent cellular cytotoxicity.

To further investigate the synergistic effect of Mosun‐Pola, we examined the activation status of T cells in vitro. To perform the investigation under conditions where the synergistic effect of Mosun‐Pola is observed, SU‐DHL‐8 target cells were pretreated with polatuzumab vedotin for 48 h. T cells and target cells were then co‐cultured and mosunetuzumab was added for a 4‐hour TDCC assay. After the assay, the cells were collected and used to measure T‐cell activation by examining the expression of CD69, an early T‐cell activation marker [22] using flow cytometry. Compared with polatuzumab vedotin or mosunetuzumab alone, the combination of Mosun‐Pola enhanced the proportion of CD69‐positive CD4^+^ and CD8^+^ T cells under the condition of the TDCC reaction against SU‐DHL‐8 cells (Figure 3A,B). Subsequently, in order to examine the effect of polatuzumab vedotin‐induced CD20 enhancement on T‐cell activation in HT cells, HT cells were pretreated with polatuzumab vedotin for 72 h and a similar experiment was performed. As a result, significant upregulation of T‐cell activation marker CD69 was also observed in HT cells (Figure 3C,D).

### 3.3 Mosun‐Pola Enhances Anti‐Tumor Activity in SU‐DHL‐8 Mouse Xenograft Model

**FIGURE 4:**
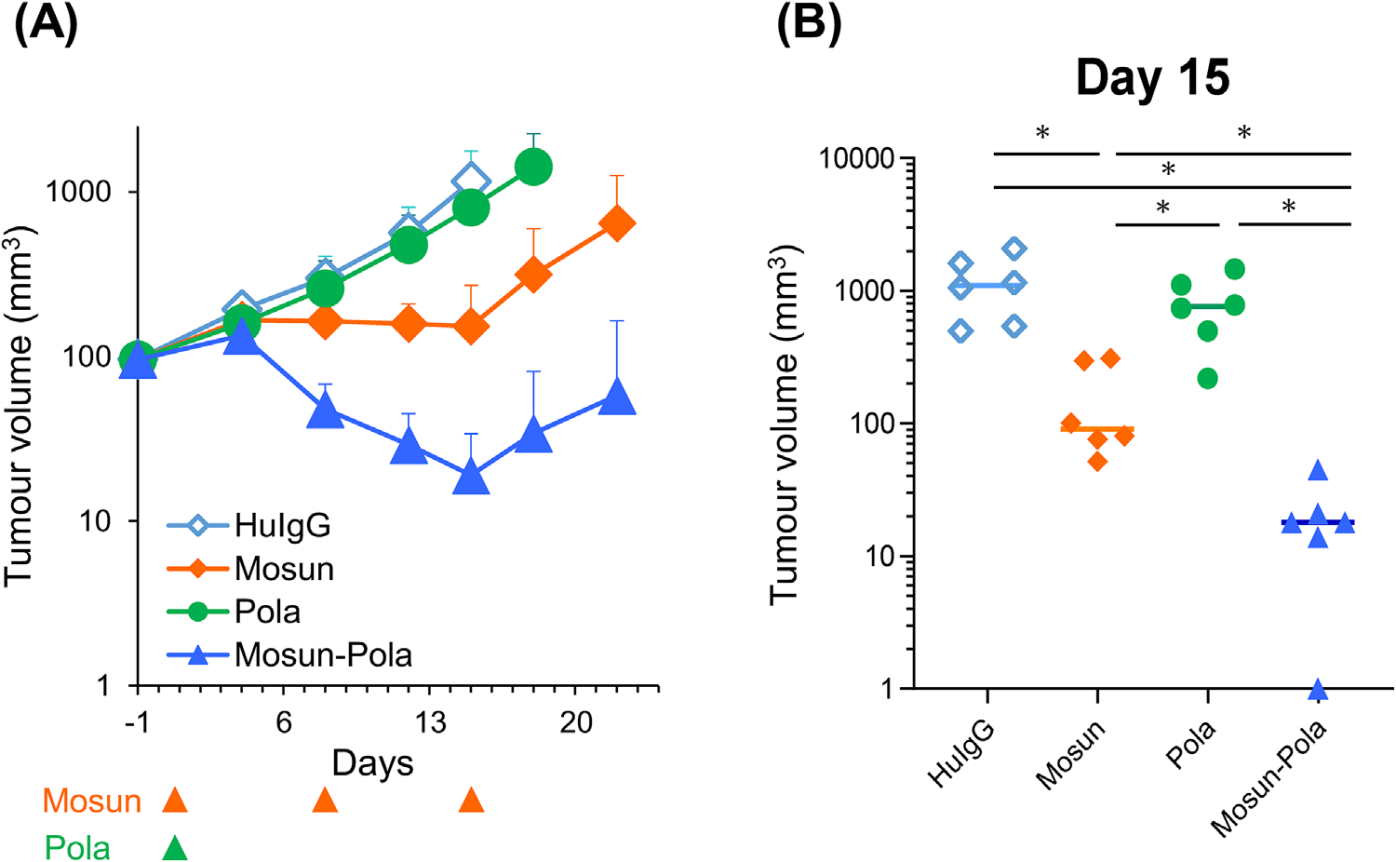
Efficacy of Mosun‐Pola in the SU‐DHL‐8 mouse xenograft model. (A) In the human T‐cell‐injected model, mosunetuzumab (0.25 mg/kg) was administered IV on Days 1, 8, and 15. Polatuzumab vedotin (2 mg/kg) was administered IV on Day 1. Tumor volume (mm^3^) is presented as mean value + SD. *n *= 6. (B) Tumor volumes measured on Day 15 are shown. Dots indicate individuals and bars represent median values. *p*‐values for the Wilcoxon rank sum test were adjusted by the Holm–Bonferroni method. *Indicates that the adjusted *p*‐value was considered statistically significant. HuIgG, human immunoglobulin G; IV, intravenously; Mosun, mosunetuzumab; Pola, polatuzumab vedotin; SD, standard deviation.

To evaluate in viv*o* anti‐tumor efficacy of Mosun ‐Pola, we employed the SU‐DHL‐8 mouse xenograft model. To address the issue of mosunetuzumab cross‐reactivity in mouse models, human T cells from PBMCs were injected IP into mice as effector cells on Day 1 (human T‐cell‐injected model) [23]. Mosunetuzumab was administered on Days 1, 8, and 15, and polatuzumab vedotin was administered on Day 1, and the study was continued to Day 22 based on the clinical dosing schedule in Cycle 1. The group treated with Mosun‐Pola showed a significantly enhanced anti‐tumor effect compared with each single‐drug group on Day 15, when tumor volume reached euthanasia criteria in the control group (HuIgG) (Figure 4A,B). We previously reported that polatuzumab vedotin induced CD20 expression in in vivo SCID mouse models [18]. To verify whether a similar effect occurs in the human T‐cell‐injected model used in this study, we evaluated CD20 expression levels in these models following polatuzumab vedotin administration. Consequently, we confirmed that polatuzumab vedotin administration significantly enhanced the amount of CD20 expression compared with the control HuIgG group in SU‐DHL‐8 tumors on Day 5 (Figure S3).

### 3.4 Enhanced Anti‐Tumor Activity of Mosun‐Pola in HT Mouse Xenograft Models

**FIGURE 5:**
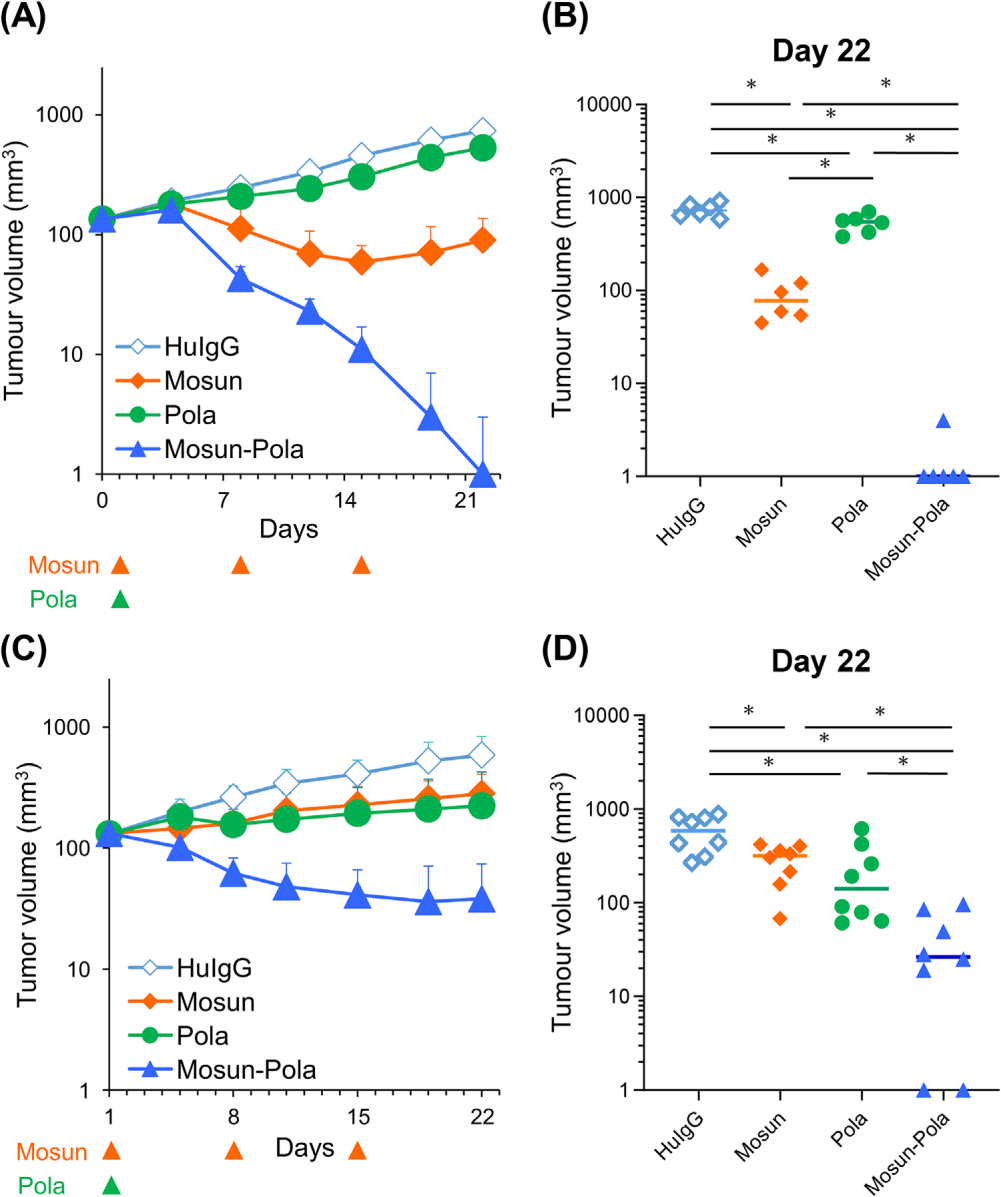
Efficacy of Mosun‐Pola in the HT mouse xenograft models. (A) In the human T‐cell‐injected model, mosunetuzumab (0.1 mg/kg) was administered IV on Days 1, 8, and 15. Polatuzumab vedotin (2 mg/kg) was administered IV on Day 1. Tumor volume (mm^3^) is presented as mean value + SD. *n *= 6. (B) Tumor volumes measured on Day 22 are shown. (C) In the huNOG mouse model, mosunetuzumab (1 mg/kg) was administered IV on Days 1, 8, and 15. Polatuzumab vedotin (2 mg/kg) was administered IV on Day 1. Tumor volume (mm^3^) is presented as mean values + SD. *n *= 8. (D) Tumor volumes measured on Day 22 are shown. (B, D) Dots indicate individuals and bars represent median values. *p*‐values for the Wilcoxon rank sum test were adjusted by the Holm–Bonferroni method. *indicates that the adjusted *p*‐value was considered statistically significant. HuIgG, human immunoglobulin G; IV, intravenously; Mosun, mosunetuzumab; Pola, polatuzumab vedotin; SD, standard deviation.

To explore the in vivo anti‐tumor combination effects in a different cell line, we subsequently evaluated the efficacy of Mosun‐Pola using a T‐cell‐injected mouse model with HT cells. On Day 22, a significant increase in anti‐tumor effect was observed in the Mosun‐Pola group compared with each single‐drug group (Figure 5A,B). We also employed a humanized mouse model, huNOG, where human T cells are differentiated and constitutively supplied through administered human CD34^+^ stem cells [24] to evaluate the combination effect of Mosun‐Pola. The Mosun‐Pola group also showed a significant increase in the anti‐tumor effect at Day 22 compared with each single‐drug group in the huNOG mouse model (Figure 5C,D), suggesting the efficacy of Mosun‐Pola in two different human immune system reconstituted mouse models.

## 4 Discussion

Mosunetuzumab, a CD20×CD3 T ‐cell‐engaging bispecific antibody that engages and redirects T cells to eliminate malignant B cells [10, 12, 16] has been reported to exhibit efficacy against lymphoma cell lines with various levels of CD20 expression [10]. On the other hand, data have been reported showing that mosunetuzumab did not demonstrate clinical activity in patients with low levels of CD20 expression [25]. Thus, many aspects regarding the relationship between CD20 expression and mosunetuzumab sensitivity remain unclear, particularly how the upregulation or downregulation of CD20 antigen expression within the cells affect the efficacy of mosunetuzumab. We previously reported that polatuzumab vedotin increases CD20 expression through the phosphorylation of ERK and AKT, enhancing sensitivity to rituximab [18]. In this study, we examined the impact of Pola‐induced CD20 upregulation on mosunetuzumab sensitivity.

**FIGURE 6:**
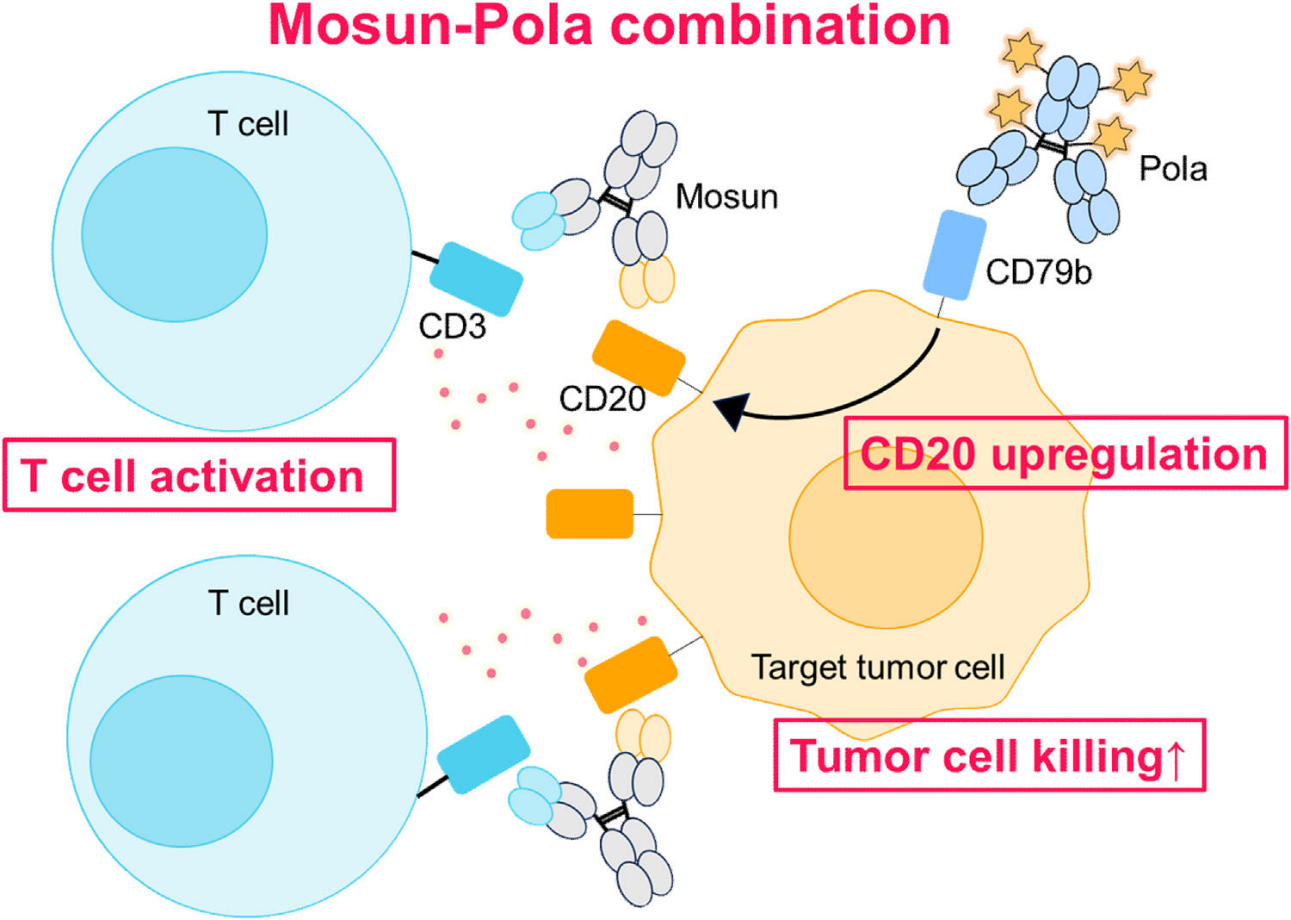
Schematic diagram of combination mechanism of polatuzumab vedotin with mosunetuzumab. Pola‐induced CD20 upregulation could contribute to the efficacy of the synergistic Mosun‐Pola combination. Mosun, mosunetuzumab; Pola, polatuzumab vedotin.

We demonstrated that Mosun‐Pola exhibits synergistic effects in SU‐DHL‐8 and HT cells, which are GCB‐type human DLBCL cell lines (Figure 6). This synergistic effect was suppressed when CD20 upregulation by polatuzumab vedotin was inhibited by AKT and ERK inhibitors, suggesting that the synergistic effect of Mosun‐Pola in vitro could be contributed to by polatuzumab vedotin‐induced CD20 upregulation, rather than by its cytotoxic effect. In other words, our findings provide the first evidence that upregulating the expression of the CD20 antigen by polatuzumab vedotin not only enhances rituximab sensitivity, which depends on CDC and ADCC, but also increases the sensitivity to mosunetuzumab, a CD20×CD3 bispecific antibody.

It has previously been demonstrated that both cell lines used in this study (SU‐DHL‐8 and HT) have low baseline sensitivity to polatuzumab vedotin [18]. These results, therefore, suggest that Mosun‐Pola could be effective even under conditions where polatuzumab vedotin sensitivity is reduced. Consequently, these results could provide supporting data for the re‐administration of polatuzumab vedotin following regimens that included polatuzumab vedotin. This study focused on two GCB‐type DLBCL cell lines; meanwhile, recent studies have reported the existence of various DLBCL genetic subtypes [26, 27]. Therefore, further investigations are required to determine if the same combination effects can be observed in tumors with various genetic backgrounds in clinical settings.

Furthermore, a previous study suggested that resistance to mosunetuzumab could be acquired through reduced CD20 transcription or mutations in clinical samples [25]. Our earlier research demonstrated that polatuzumab vedotin enhances CD20 mRNA expression. Thus, the combination of Mosun‐Pola might overcome resistance to mosunetuzumab caused by decreased CD20 mRNA expression. In addition, in cases where CD20 mutations have resulted in reduced affinity to mosunetuzumab, increasing surface CD20 expression might reverse this resistance. Further studies are necessary to explore these possibilities.

Previous research has indicated the importance of both CD4^+^ and CD8^+^ T cells in mosunetuzumab‐mediated tumor killing [10]. To gain more insight into the synergistic effects observed with Mosun‐Pola, we analyzed the expression of T‐cell activation markers in these cell populations. Specifically, to investigate the possibility that increased expression of the CD20 antigen leads to a faster rise in T‐cell activation, we used CD69, an early activation marker, and evaluated T‐cell activation status at an early point (4 h) after initiating the TDCC reaction. Our results showed that Mosun‐Pola significantly elevated the proportions of CD69^+^CD4^+^ and CD69^+^CD8^+^ cells. Whilst further studies are needed to elucidate the detailed mechanism, we hypothesize that upregulated CD20 antigen expression may have enhanced T‐cell target cell recognition efficiency, leading to more effective immune synapse formation and T‐cell activation.

For conducting combination studies in vivo, we utilized two mouse models reconstituted with human immune cells to overcome the difficulties of mosunetuzumab cross‐reactivity in mouse models. The first model, which involved the injection of in vitro activated human T cells into NOD/SCID mice (T‐cell‐injected mouse model), offers the advantage of directly evaluating T‐cell contribution to the combination efficacy. The second was the huNOG mouse model, which reconstructs the human immune system through hematopoietic stem cell transplantation. We consider this huNOG mouse model to be closer to physiological conditions because it enables the evaluation of immune cell interactions reconstructed and maintained in vivo. Although both mouse models have limitations due to limited immune cell diversity and artificially constructed immune environments, significantly enhanced anti‐tumor effects were detected with Mosun‐Pola, suggesting the potential efficacy of this combination therapy in vivo. During the in vivo study, we used a schedule in which mosunetuzumab and polatuzumab vedotin were co‐administered on Day 1, following the design of the SUNMO trial [17]. In this study, we confirmed that polatuzumab vedotin showed CD20 upregulation effects on Day 5, after treatment. In addition, our previous study has shown significant CD20 upregulation by polatuzumab vedotin on Day 14 post‐treatment [18]. These findings indicate that polatuzumab vedotin‐mediated CD20 upregulation is sustained rather than transient, and suggest some flexibility in the time during which the Mosun‐Pola combination shows efficacy. Therefore, we consider that co‐administering both drugs on the same day allows adequate time for CD20 upregulation to occur and enhance the therapeutic effect of the combination.

A recent study conducting a comprehensive analysis of the DLBCL immune micro‐environment showed that tumors classified as “immune‐hot” were associated with enhanced clinical efficacy of mosunetuzumab monotherapy compared with “immune‐cold” environments in clinical settings [28]. In our current investigation, we found that Mosun‐Pola promotes T‐cell activation. Furthermore, our previous research has shown that polatuzumab vedotin affects the tumor micro‐environment, including the innate immune system [29]. Accordingly, future investigations in clinical settings are needed to determine whether the combination of Mosun‐Pola modulates the tumor micro‐environment in DLBCL, as well as to elucidate in detail how this micro‐environment influences the efficacy of this combination therapy.

In conclusion, our findings present a novel rationale for the Mosun‐Pola combination in both in vitro and in vivo DLBCL models. Our findings suggest that polatuzumab vedotin upregulates the expression of CD20, leading to increased sensitivity to mosunetuzumab and the potential to overcome resistance to this therapy. Furthermore, Mosun‐Pola may affect the immune micro‐environment in DLBCL, potentially influencing the efficacy of this combination therapy. We believe that this study could provide evidence supporting the clinical importance of this regimen.

## Author Contributions

Natsumi Kawasaki, Sei Shu, Mayu Tomita and Yoriko Yamashita‐Kashima conceived the idea, designed and performed the experiments, analysed the data and wrote the manuscript. Xiaoxiao Liu conceived the idea, designed and performed the experiments, and analysed the data. Shigeki Yoshiura and Yoriko Yamashita‐Kashima supervised this study. All authors contributed to the final manuscript and approved it for submission.

## Ethics Statement

This study used commercially available human PBMCs that were obtained with proper ethical approval.

## Consent

The authors have nothing to report.

## Conflicts of Interest

All authors are employees of Chugai Pharmaceutical Co., Ltd. Writing of this manuscript was supported by F. Hoffmann‐La Roche, Ltd.

## Supporting information




**Supporting Figure S1**: Surface expression of CD20 on SU‐DHL‐8 cells transduced with either non‐targeting shRNA (shControl) or CD20‐targeting shRNA (shCD20#1) was measured by flow cytometry and dot plots are shown.

**FigureS2**: The effect of ERK and AKT inhibition on polatuzumab vedotin‐induced upregulation of CD20 expression.

**FigureS3**: The effect of polatuzumab vedotin on CD20 upregulation in SU‐DHL‐8 mouse xenograft model.

## Data Availability

The data that support the findings of this study are available from the corresponding author upon reasonable request.
