# Supplementary figures and images for: Polatuzumab Vedotin Induced CD20 Upregulation Contributes to the Efficacy of Mosunetuzumab in Combination With Polatuzumab Vedotin in Diffuse Large B‐Cell Lymphoma Preclinical Models
Source: EJHaem. 2025 Oct 24;6(5):e70169. doi: 10.1002/jha2.70169 (PMC12551598; doi:10.1002/jha2.70169)

shControl

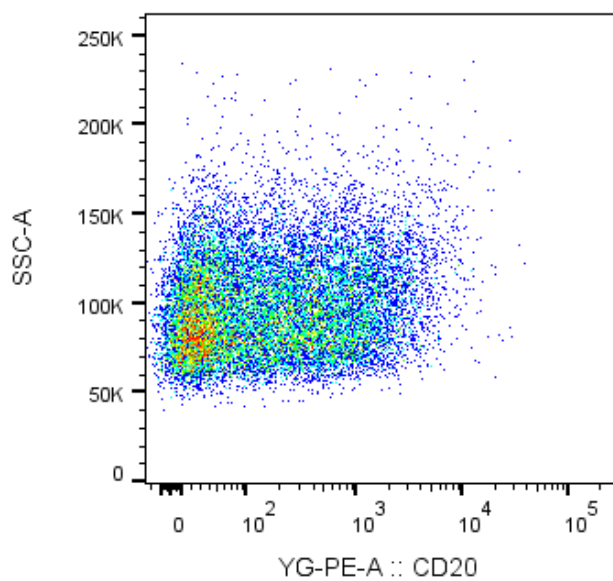

shCD20#1

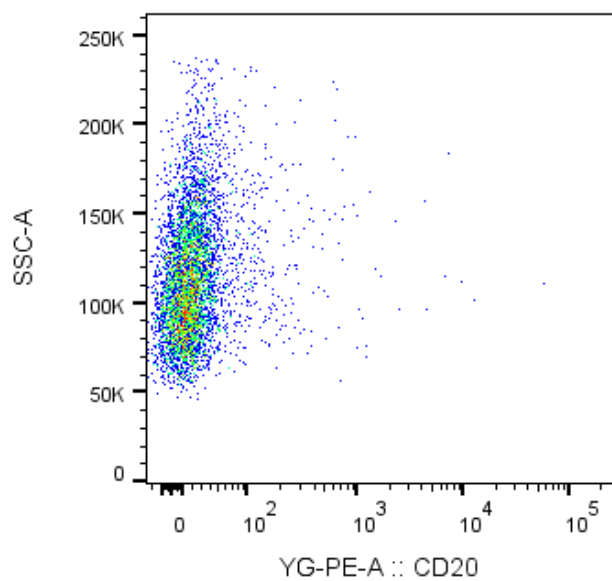

Supplement: Supplementary file 1 — Supporting Figure S1: Surface expression of CD20 on SU‐DHL‐8 cells transduced with either non‐targeting shRNA (shControl) or CD20‐targeting shRNA (shCD20#1) was measured by flow cytometry and dot plots are shown. [file JHA2-6-e70169-s003.pdf]

**(A)**

SU-DHL-8  
CD20

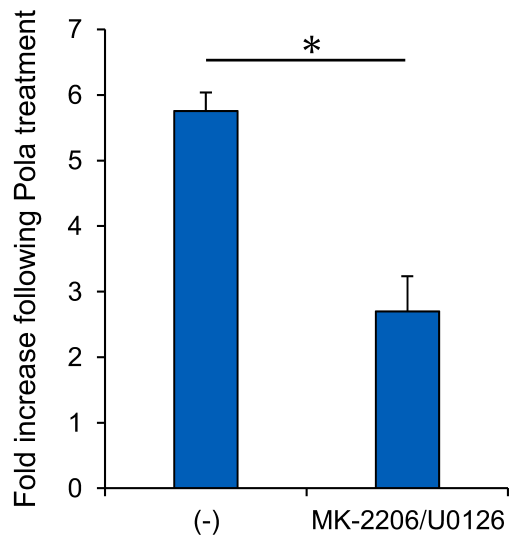

**(B)**

HT  
CD20

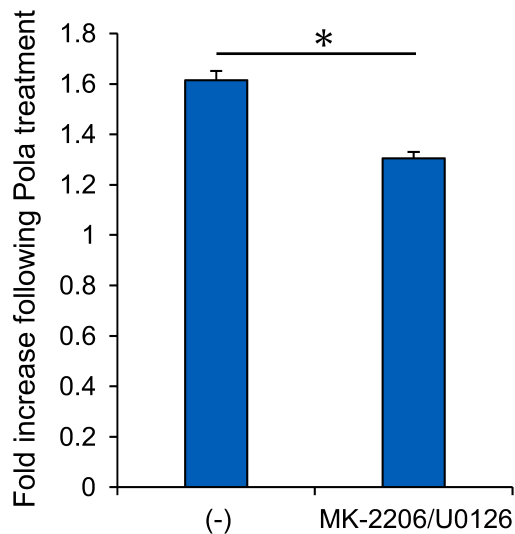

Supplement: Supplementary file 2 — FigureS2: The effect of ERK and AKT inhibition on polatuzumab vedotin‐induced upregulation of CD20 expression. [file JHA2-6-e70169-s001.pdf]

## CD20

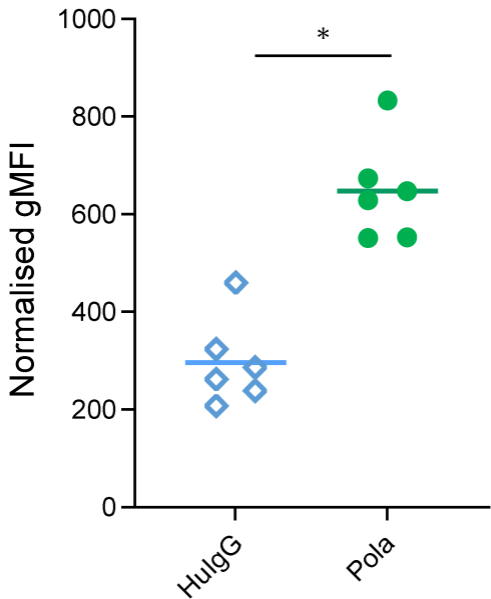

Supplement: Supplementary file 3 — FigureS3: The effect of polatuzumab vedotin on CD20 upregulation in SU‐DHL‐8 mouse xenograft model. [file JHA2-6-e70169-s002.pdf]
